# Supplementary material for: SYPL1 defines a vesicular pathway essential for sperm cytoplasmic droplet formation and male fertility
Source: Nat Commun. 2023 Aug 22;14:5113. doi: 10.1038/s41467-023-40862-1 (PMC10444883; doi:10.1038/s41467-023-40862-1)
Supplement: Supplementary file 1 — Supplementary Information [file 41467_2023_40862_MOESM1_ESM.pdf]

## Supplementary information

Supplementary information includes:

- Supplementary Figures. 1-10
- Supplementary Tables. 1-3

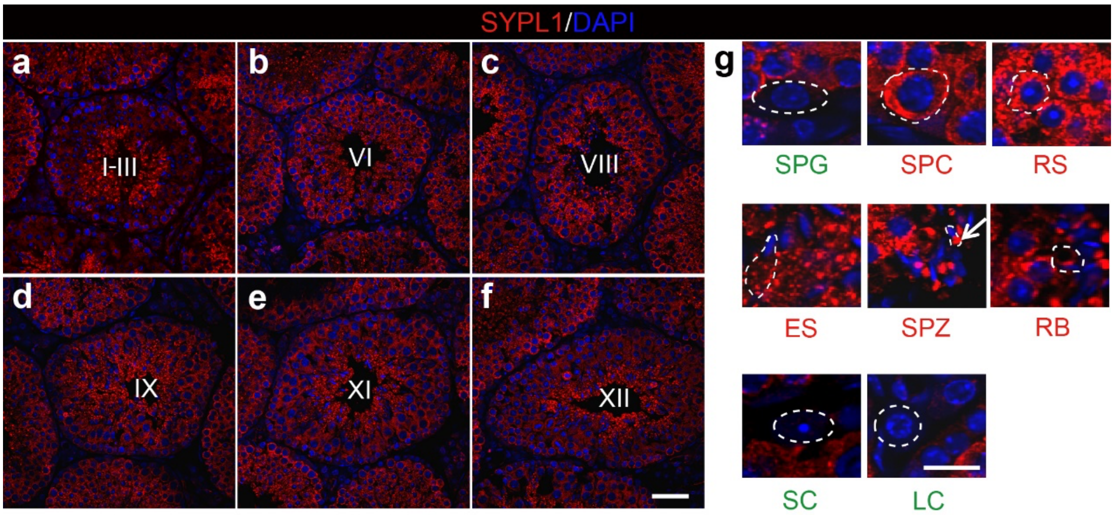


**Supplementary Figure 1 (Fig. S1). SYPL1 expression in the mouse testis**. **a-f** SYPL1 expression in different stages of seminiferous tubules by immunofluorescence. Scale bar, 50μm. **g** Immunofluorescence showing SYPL1 localization in SPC, RS, ES, SPZ and RB, but undetectable in SPG, SC and LC of WT testis. SPC, spermatocyte; RS, round spermatid; ES, elongating/elongated spermatid; SPZ, spermatozoon; RB, residual body; SPG, spermatogonium; SC, Sertoli cell; LC, Leydig cell. A white arrow indicates CD. Scale bar, 20μm.

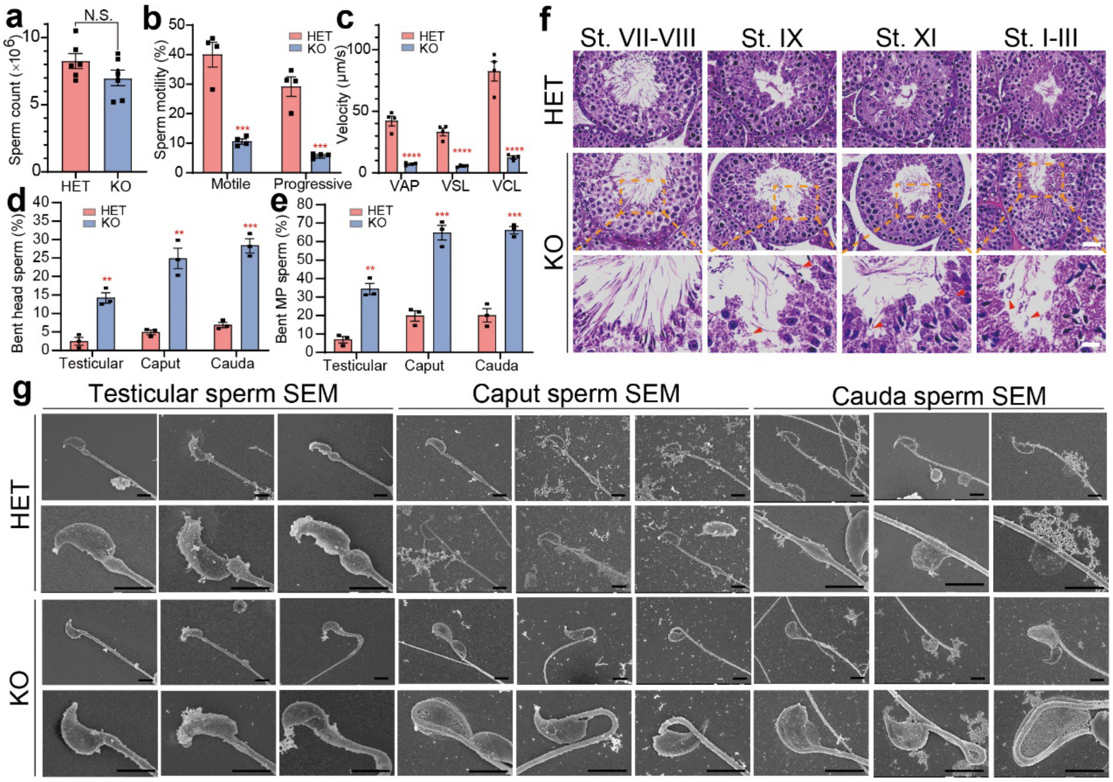


**Fig. S2. Morphological and motility defects in KO sperm. a** Sperm count showing no significant difference in the sperm number of cauda epididymides in HET and KO mice. Biologically independent mice (n=6) were examined in this experiment. Data are presented as mean ± SEM. A two-sided Student's *t*-test was performed. N.S., no significant difference, $p$=0.153. **b** The proportion of motile and progressive sperm in the cauda epididymis of HET and KO mice. Biologically independent mice (n=4) were examined in this experiment. Data are presented as mean ± SEM. A two-sided Student's *t*-test was performed, left to right: *** $p=3.64\times10^{-4}$, *** $p=3.30\times10^{-4}$. **c** Evaluation of sperm motility velocity. VAP, average path velocity, VSL, straight-line velocity, VCL, curvilinear velocity. Biologically independent mice (n=4) were examined in this experiment. Data are presented as mean ± SEM. A two-sided Student's *t*-test was performed, left to right: **** $p=8.94\times10^{-5}$, **** $p=6.21\times10^{-5}$, ****$p=9.83\times10^{-5}$. **d** Percentages of bent head sperm. Biologically independent mice (n=3) were examined in this experiment. Data are presented as mean ± SEM. A two-sided Student's *t*-test was performed, left to right: **$p=2.21\times10^{-3}$, **$p=2.09\times10^{-3}$, ***$p=3.28\times10^{-4}$. **e** Percentages of bent midpiece sperm. Biologically independent mice (n=3) were examined in this experiment. Data are presented as mean ± SEM. A two-sided Student's *t*-test was performed, left to right: **$p=1.33\times10^{-3}$, ***$p=7.14\times10^{-4}$, ***$p=3.18\times10^{-4}$. **f** Histology of HET and KO testes. Red arrowheads indicate angulated sperm. St., stage; scale bar, 30μm (upper and middle panels) and 10μm (down enlarged panel). **g** Scanning electron microscopy (SEM) of *Sypl1* KO and HET isolated testicular and epididymal sperm. Scale bar, 5μm. Source data are provided as a Source Data file.

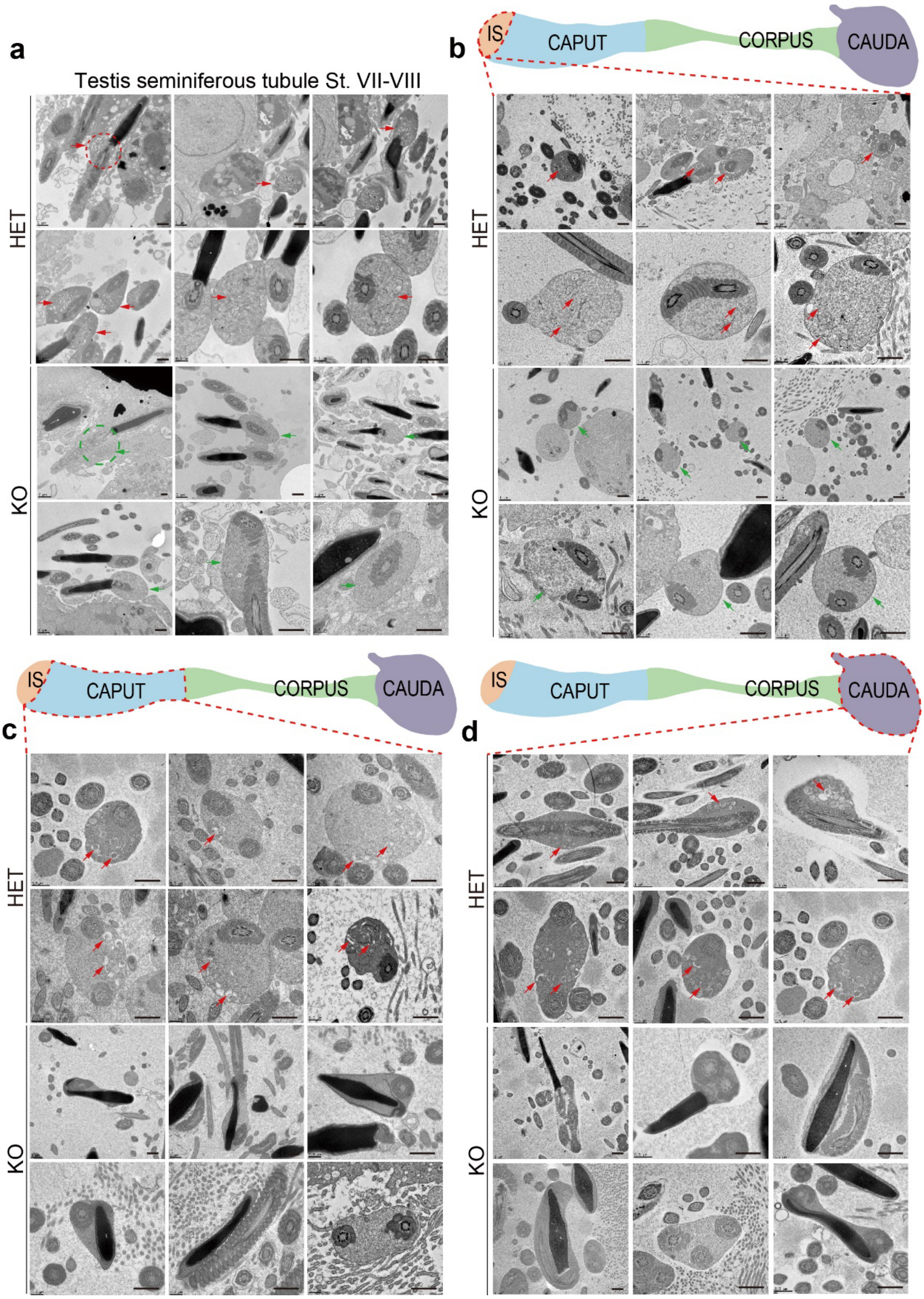


**Fig. S3. Loss of saccular elements in the CD region of testicular spermatozoa and epididymal sperm in KO mice. a** TEM analyses of stage VII-VIII seminiferous tubules. Red dotted circle indicates the CD region and saccular elements in HET testicular spermatozoa before release. Red arrows indicate saccular elements in the CD region of HET testicular spermatozoa. Green dotted circle indicates the CD region and absence of saccular elements in KO testicular spermatozoa before

release. Green arrows indicate loss of typical saccular elements in the CD region of KO testicular spermatozoa. St., stage; scale bar, 1μm**.** **b** TEM analyses showing the loss of saccular elements in the CD region of IS (initial segment) epididymal sperm in KO mice. Red arrows indicate saccular elements in the CD region of HET IS sperm. Green arrows indicate loss of typical saccular elements in the CD region of KO IS sperm. Scale bar, 1μm. **c** TEM analyses showing the absence of saccular elements in the CD region of caput epididymal sperm in KO mice. Red arrows indicate saccular elements in the CD region of HET caput sperm. Scale bar, 1μm. **d** TEM analyses showing the absence of saccular elements in the CD region of cauda epididymal sperm in KO mice. Red arrows indicate saccular elements in the CD region of HET caput sperm. Scale bar, 1μm.

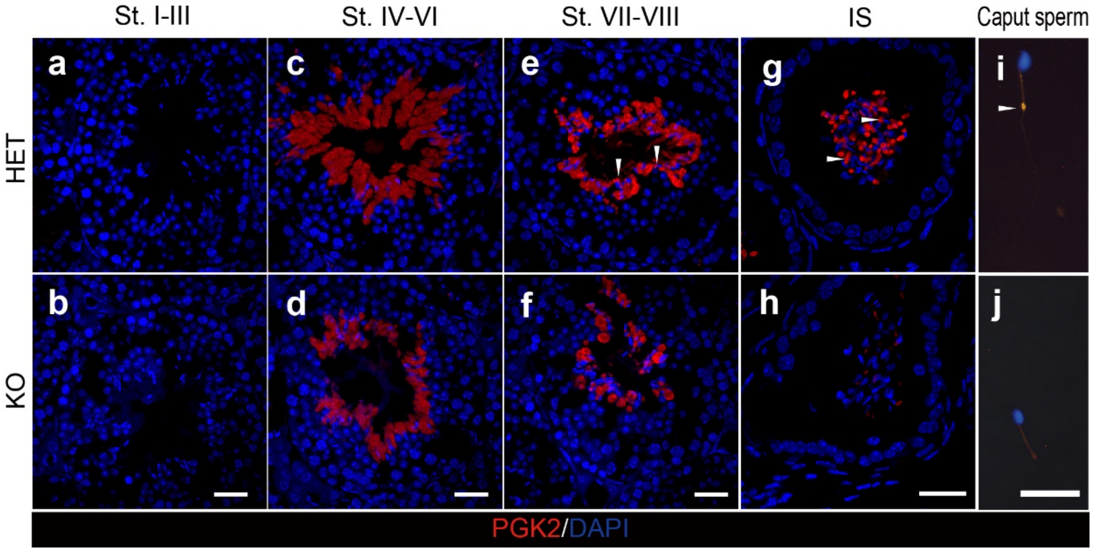


**Fig. S4.** ***Sypl1*** **deletion leads to loss of PGK2 in the sperm CD. a-j** Immunofluorescence of PGK2 in testes, IS epididymis, and caput sperm of HET and KO mice. PGK2 signals in the CD regions are indicated by white arrowheads. IS, initial segment; St., stage; scale bar, 30μm in **a-h**, 20μm in **i** and **j.**

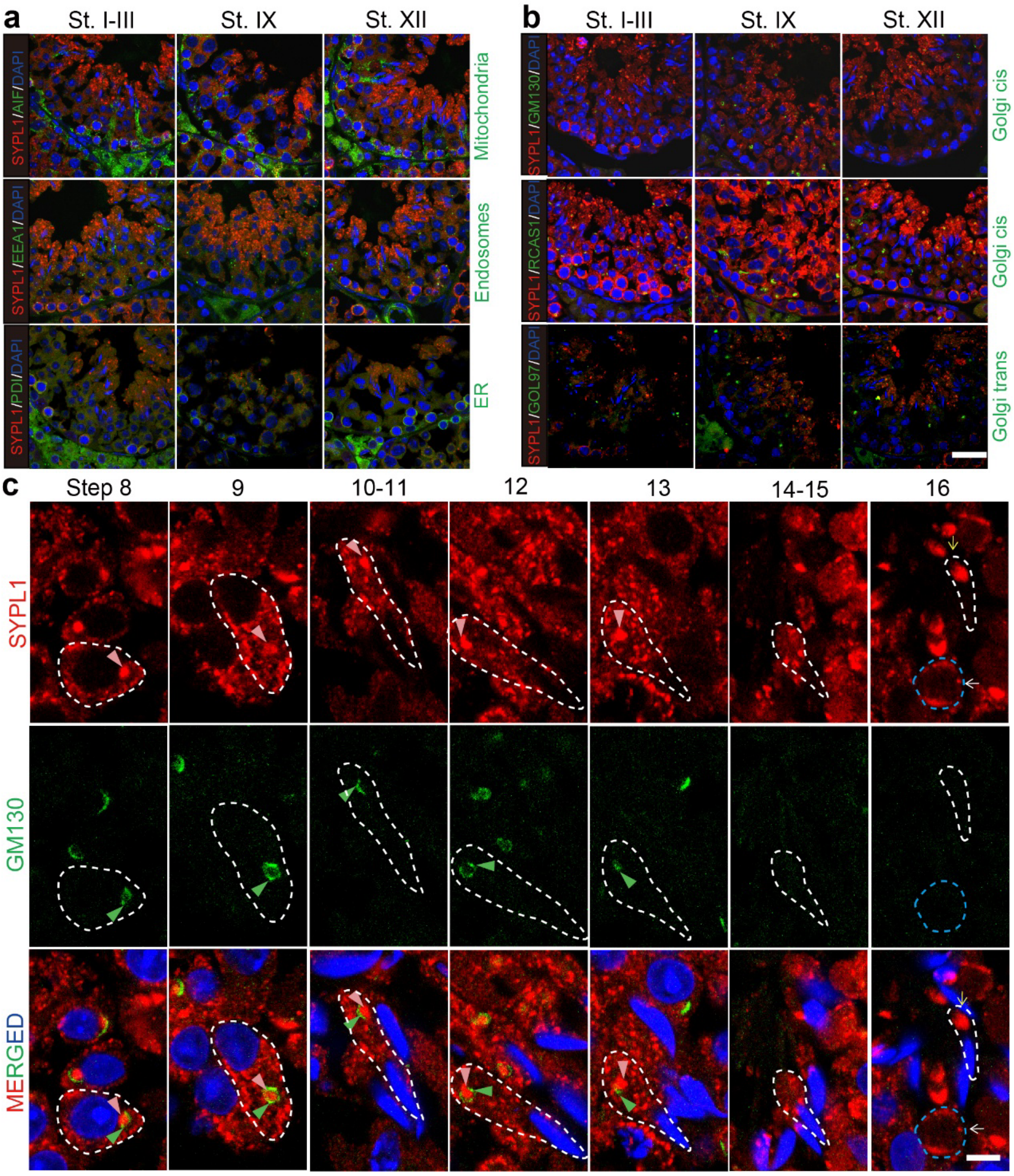


**Fig. S5. Co-staining of SYPL1 with organelle markers. a** and **b** in stage I-II, IX and XII seminiferous tubules of WT mouse testis. St., stage; scale bar, 30μm. **c** Co-localization of SYPL1 and GM130, a cis Golgi network marker. Pink arrowheads indicate SYPL1 and green arrowheads show the cis Golgi network. Yellow arrows indicate the CD and white arrows indicate the residual body (circled by blue dotted lines). Scale bar, 5μm.

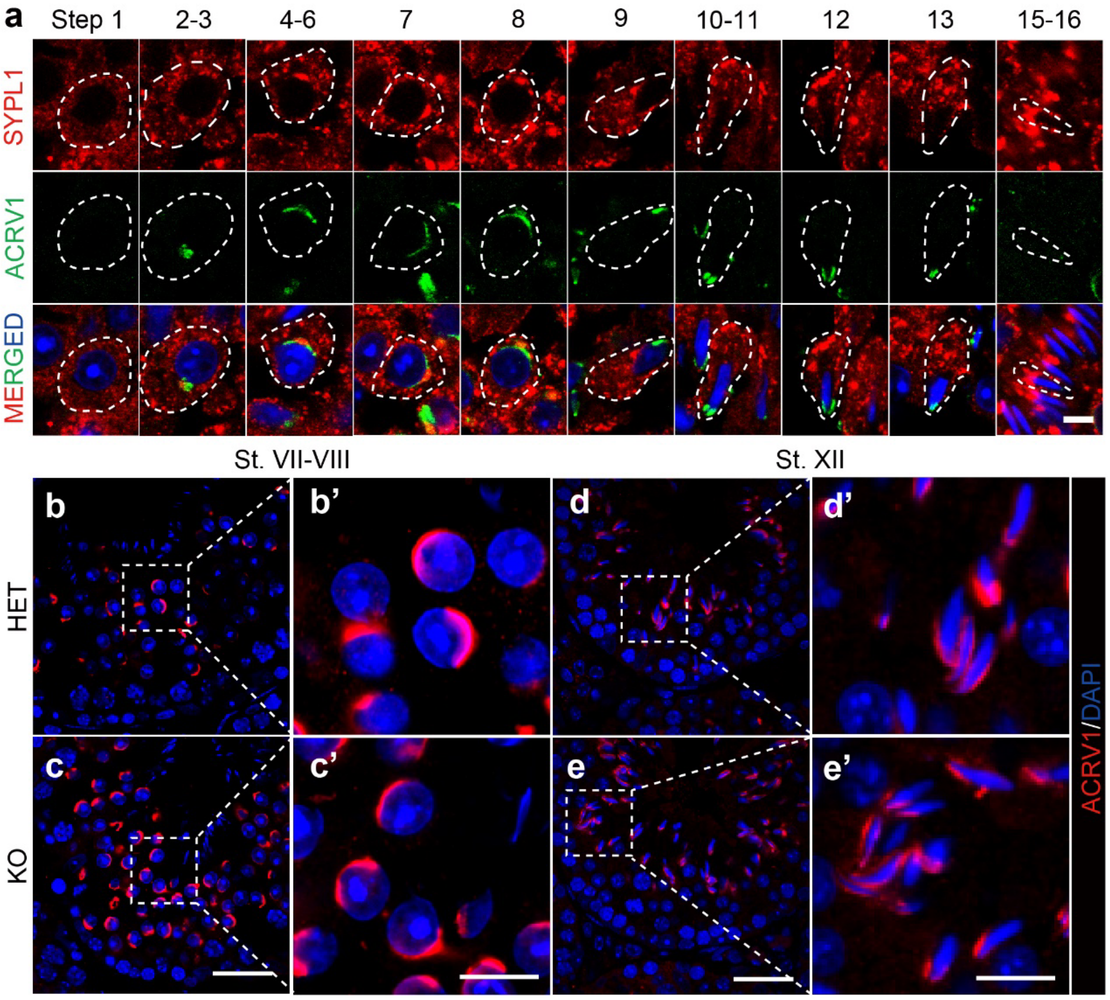


**Fig. S6. *Sypl1* deletion does not affect the formation of acrosomes. a** Co-localization of SYPL1 and ACRV1, an acrosome marker, in WT step1-16 spermatids. Open dotted white lines indicate the borders of spermatids. Scale bar, 5μm. **b-e'** Immunofluorescence of ACRV1 in seminiferous tubules of HET and KO testes. St., stage; scale bar, 30μm in **b**, **c**, **d** and **e**, and 10μm in **b'**, **c'**, **d'** and **e'.**

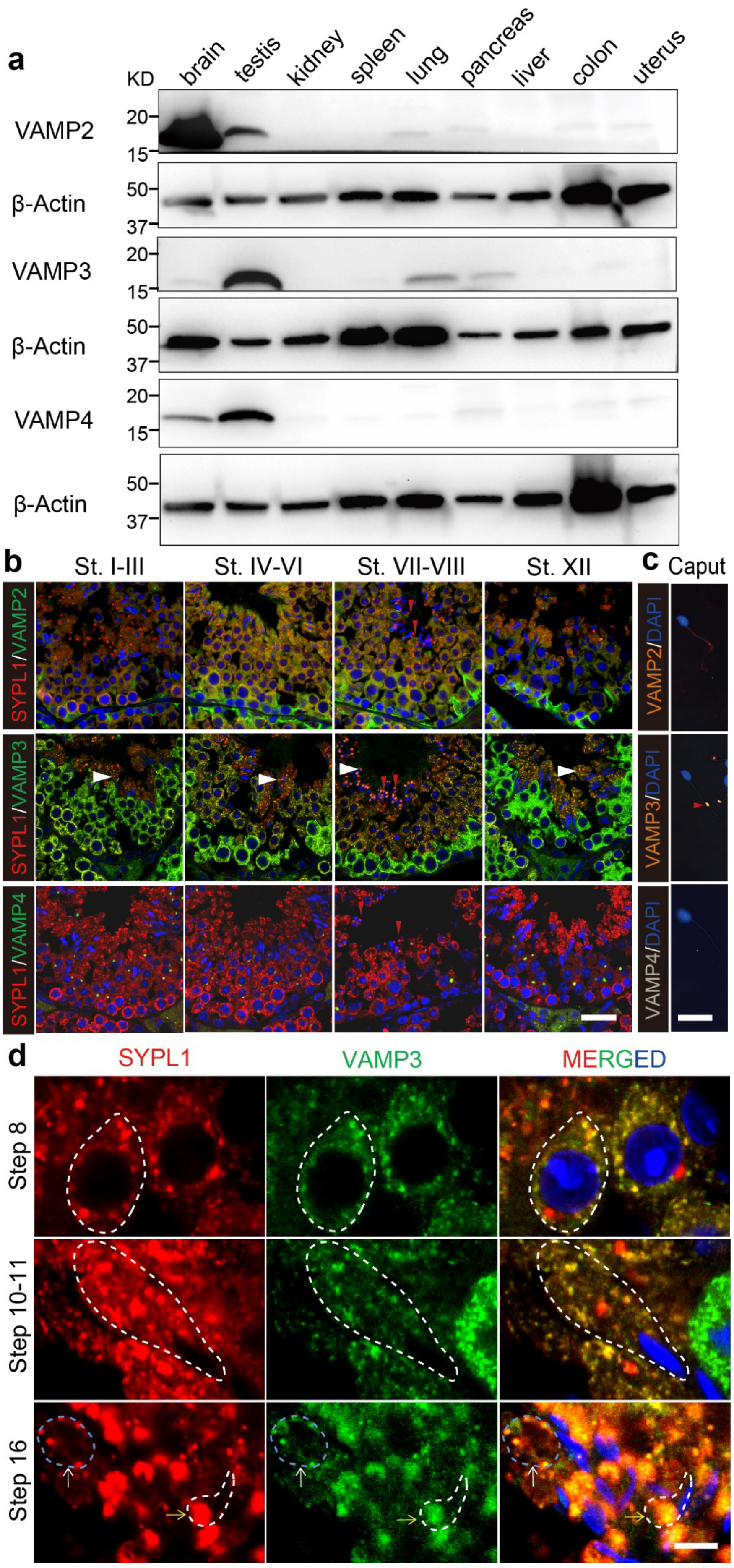
a
KD
brain
testis
kidney
spleen
lung
pancreas
liver
colon
uterus
VAMP2
β-Actin
VAMP3
β-Actin
VAMP4
β-Actin
20
15
50
37
b
St. I-III
St. IV-VI
St. VII-VIII
St. XII
SYPL1/VAMP2
SYPL1/VAMP3
SYPL1/VAMP4
c
Caput
VAMP2/DAPI
VAMP3/DAPI
VAMP4/DAPI
d
SYPL1
VAMP3
MERGED
Step 8
Step 10-11
Step 16

**Fig. S7. Co-staining of SYPL1 with VAMP family proteins. a** Tissue expression of VAMP2, VAMP3 and VAMP4 in mice by Western blots. β-actin serves as a loading control. **b** Co-staining of SYPL1 with VAMP2, VAMP3 and VAMP4 in WT mouse testes. White arrowheads indicate the co-localization of SYPL1 and VAMP3 in elongating/elongated spermatids. St., stage; scale bar, 30μm. **c** Immunofluorescence of VAMP2, VAMP3 and VAMP4 in mouse caput epididymal sperm. The red arrowhead indicates VAMP3 signals in the CD. Scale bar, 20μm. **d** Co-localization of SYPL1 and VAMP3 in step 8-16 spermatids of WT testes. Dotted white lines indicate the borders of different spermatids. Yellow arrowheads indicate CDs and white arrowheads indicate the residual bodies (circled by blue dotted lines). Scale bar, 5μm. Source data are provided as a Source Data file.

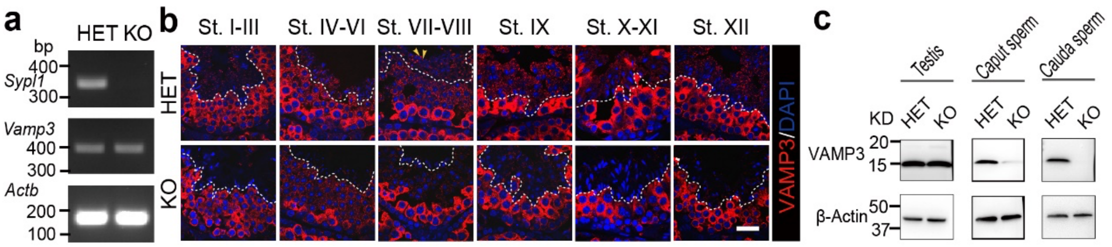


**Fig. S8. The effect of SYPL1 deficiency on VAMP3 expression and localization. a** RT-PCR showing *Sypl1* and *Vamp3* mRNA in HET and KO testes. β-Actin serves as an internal control. **b** Immunofluorescence of VAMP3 in seminiferous tubules of HET and KO testes. Positive VAMP3 signal in CDs are indicated by yellow arrowheads. Open dotted white lines indicate the borders of different step spermatids (step 9-16). St., stage; scale bar, 30μm. **c** Western blotting showing VAMP3 protein levels in testes, caput sperm and cauda sperm from HET and KO mice. β-Actin serves as a loading control. Source data are provided as a Source Data file.

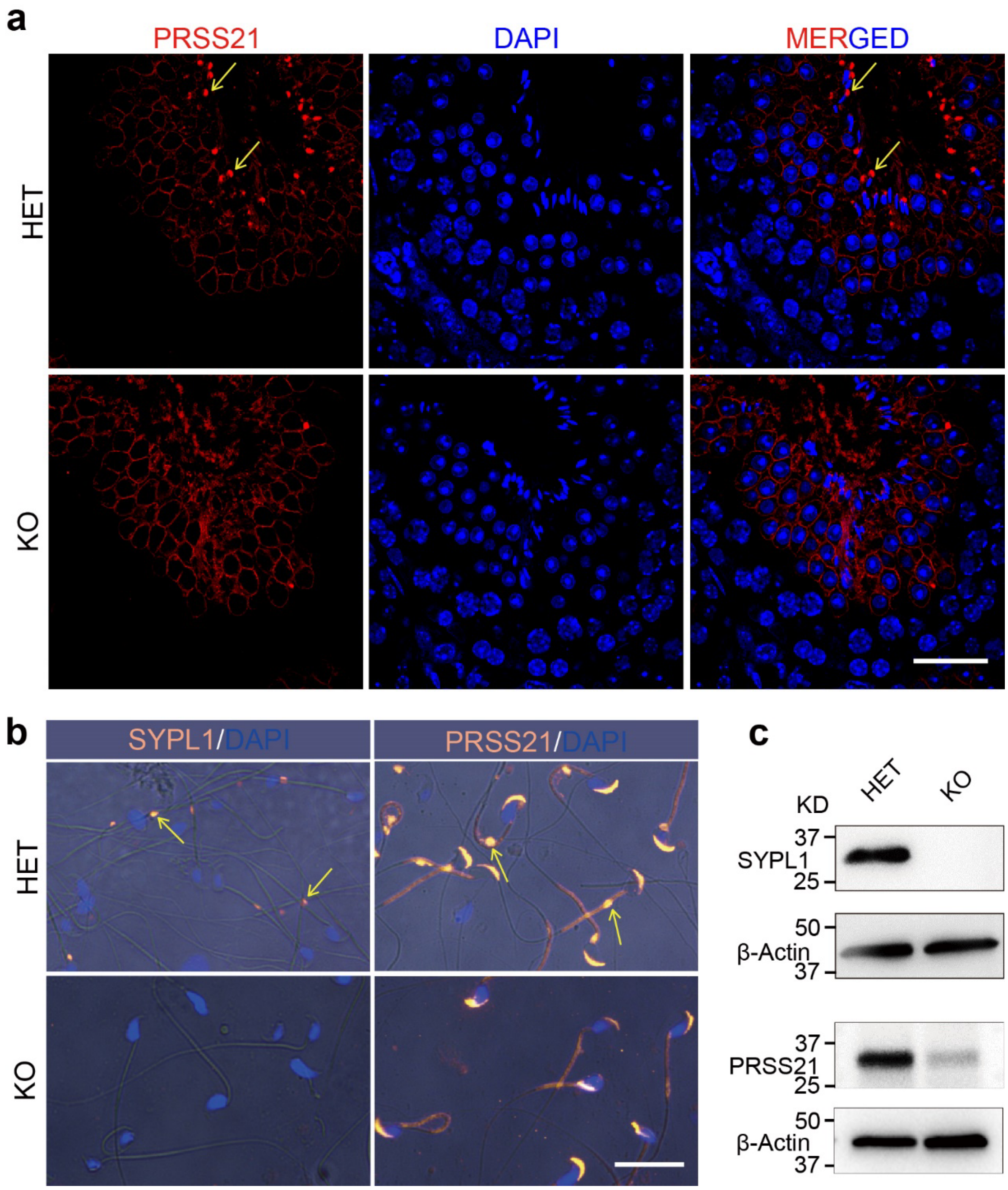


**Fig. S9**. **SYPL1 deficiency leads to the loss of PRSS21 in the CD. a** Immunofluorescence of PRSS21 in HET and KO testes. Yellow arrows indicate PRSS21 signals in CDs. Scale bar, 30μm. **b** Immunofluorescence of SYPL1 and PRSS21 in HET and KO caput sperm. Yellow arrows indicating positive signal in CDs. Scale bar, 20μm. **c** Western blotting of PRSS21 in HET and KO caput sperm. β-Actin serves as a loading control. Source data are provided as a Source Data file.

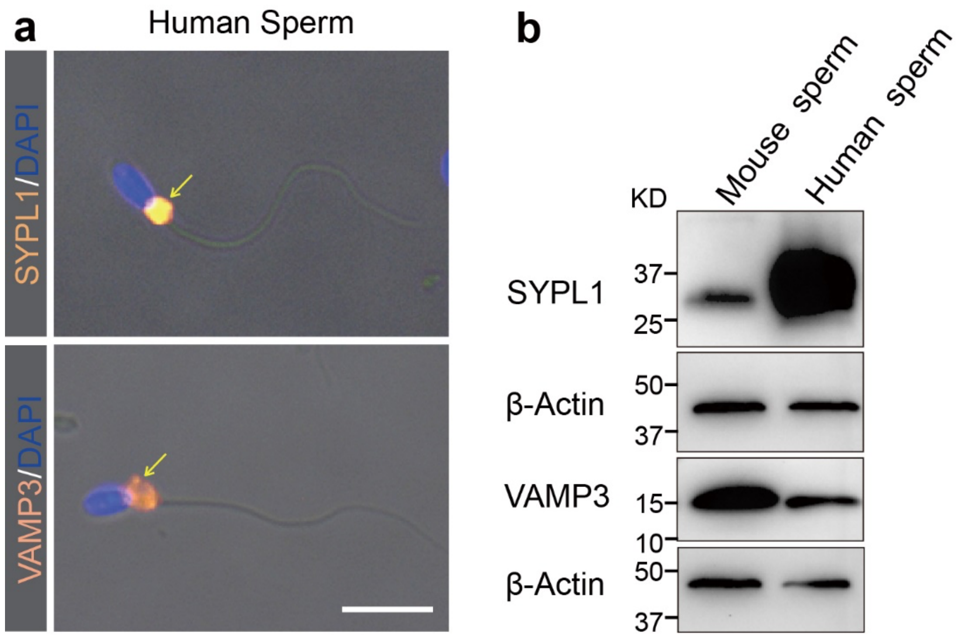


**Fig. S10. Expression of SYPL1 and VAMP3 in human sperm. a** Immunofluorescence of SYPL1 and VAMP3 in CDs of human sperm. Yellow arrows indicate positive signals in CDs. Scale bar, 10μm. **b** Western blotting of SYPL1 and VAMP3 in mouse and human sperm. β-Actin serves as a loading control. Source data are provided as a Source Data file.

**Supplementary Table 1 (Table S1). Fertility tests within 5 months.** Data are presented as mean ± SEM. A two-sided Student's *t*-test was performed, **** $P$=7.262×$10^{-12}$, ****$P$=7.02×$10^{-5}$ for litter size and litter interval, respectively. Source data are provided as a Source Data file.

| | ♂HET (N=3) × ♀WT | ♂HET (N=4) × ♀HET | ♂HET (N=4) × ♀KO | ♂KO (N=10) × ♀WT |
|---|---|---|---|---|
| Litter size (pups/litter) | 6.51±0.18 | 6.43±0.28 | 6.29±0.45 | ≈0****<br>8 male KO: no pups<br>1 male KO: 1 litter of 2 pups<br>1 male KO: 1 litter of 5 pups |
| Litter interval (Litter/month) | 1.13±0.06 | 1.15±0.05 | 1.15±0.05 | ≈0**** |

**Table S2. Percentages of epididymal sperm bearing typical large dilated CD regions by phase contrast microscope.** Biologically independent mice (n=3) were examined in this experiment. Data are presented as mean ± SEM. A two-sided Student's *t*-test was performed, $****P=5.21\times10^{-6}$, $****P=9.61\times10^{-7}$ for caput epididymal sperm and cauda epididymal sperm, respectively. Source data are provided as a Source Data file.

| | HET (%) | KO (%) |
|---|---|---|
| Caput epididymal sperm | 91.6±2.8 | 0**** |
| Cauda epididymal sperm | 86.87±1.73 | 0**** |

**Table S3. Percentages of sperm bearing typical saccular elements in the CD region by TEM.** Biologically independent mice (n=3) were examined in this experiment. Data are presented as mean ± SEM. A two-sided Student's *t*-test was performed, $****P = 1.23\times10^{-7}$, $****P = 1.45\times10^{-8}$ for testicular sperm and caput epididymal sperm, respectively. Source data are provided as a Source Data file.

| | HET (%) | KO (%) |
|---|---|---|
| Testicular sperm (Step 16) | 97.92±2.08 | 0**** |
| Caput epididymal sperm | 91.33±0.64 | 0**** |
