# Supplementary material for: SYPL1 defines a vesicular pathway essential for sperm cytoplasmic droplet formation and male fertility
Source: Nat Commun. 2023 Aug 22;14:5113. doi: 10.1038/s41467-023-40862-1 (PMC10444883; doi:10.1038/s41467-023-40862-1)
Supplement: Supplementary file 3 — Reporting Summary [file 41467_2023_40862_MOESM3_ESM.pdf]

Corresponding author(s): Chen Chen

Last updated by author(s): 8/9/2023

Reporting Summary

Nature Portfolio wishes to improve the reproducibility of the work that we publish. This form provides structure for consistency and transparency in reporting. For further information on Nature Portfolio policies, see our Editorial Policies and the Editorial Policy Checklist.

Statistics

For all statistical analyses, confirm that the following items are present in the figure legend, table legend, main text, or Methods section.

n/a	Confirmed	
☐	☒	The exact sample size (*n*) for each experimental group/condition, given as a discrete number and unit of measurement
☐	☒	A statement on whether measurements were taken from distinct samples or whether the same sample was measured repeatedly
☐	☒	The statistical test(s) used AND whether they are one- or two-sided<br>*Only common tests should be described solely by name; describe more complex techniques in the Methods section.*
☒	☐	A description of all covariates tested
☒	☐	A description of any assumptions or corrections, such as tests of normality and adjustment for multiple comparisons
☐	☒	A full description of the statistical parameters including central tendency (e.g. means) or other basic estimates (e.g. regression coefficient) AND variation (e.g. standard deviation) or associated estimates of uncertainty (e.g. confidence intervals)
☐	☒	For null hypothesis testing, the test statistic (e.g. *F*, *t*, *r*) with confidence intervals, effect sizes, degrees of freedom and *P* value noted<br>*Give P values as exact values whenever suitable.*
☒	☐	For Bayesian analysis, information on the choice of priors and Markov chain Monte Carlo settings
☒	☐	For hierarchical and complex designs, identification of the appropriate level for tests and full reporting of outcomes
☒	☐	Estimates of effect sizes (e.g. Cohen's *d*, Pearson's *r*), indicating how they were calculated

Our web collection on statistics for biologists contains articles on many of the points above.

Software and code

Policy information about availability of computer code

Data collection	N/A
Data analysis	N/A

For manuscripts utilizing custom algorithms or software that are central to the research but not yet described in published literature, software must be made available to editors and reviewers. We strongly encourage code deposition in a community repository (e.g. GitHub). See the Nature Portfolio guidelines for submitting code & software for further information.

Data

Policy information about availability of data

All manuscripts must include a data availability statement. This statement should provide the following information, where applicable:

- Accession codes, unique identifiers, or web links for publicly available datasets
- A description of any restrictions on data availability
- For clinical datasets or third party data, please ensure that the statement adheres to our policy

All data that support the findings of this study are available within the article and its supplementary information files or source data. Source data are provided in this paper.

Research involving human participants, their data, or biological material

Policy information about studies with human participants or human data. See also policy information about sex, gender (identity/presentation), and sexual orientation and race, ethnicity and racism.

Reporting on sex and gender	N/A
Reporting on race, ethnicity, or other socially relevant groupings	N/A
Population characteristics	N/A
Recruitment	N/A
Ethics oversight	N/A

Note that full information on the approval of the study protocol must also be provided in the manuscript.

Field-specific reporting

Please select the one below that is the best fit for your research. If you are not sure, read the appropriate sections before making your selection.

☒ Life sciences ☐ Behavioural & social sciences ☐ Ecological, evolutionary & environmental sciences

For a reference copy of the document with all sections, see nature.com/documents/nr-reporting-summary-flat.pdf

Life sciences study design

All studies must disclose on these points even when the disclosure is negative.

Sample size	Sample size=3-10. The sample size was determined by the number of animals available to ensure biological reproducibility and statistical significance.
Data exclusions	No data were excluded for analyses.
Replication	All experiments were performed in at least 3 replicates to ensure reproducibility and all attempts at replication were successful.
Randomization	N/A
Blinding	N/A

Reporting for specific materials, systems and methods

We require information from authors about some types of materials, experimental systems and methods used in many studies. Here, indicate whether each material, system or method listed is relevant to your study. If you are not sure if a list item applies to your research, read the appropriate section before selecting a response.

Materials & experimental systems

n/a	Involved in the study	
☐	☒	Antibodies
☒	☐	Eukaryotic cell lines
☒	☐	Palaeontology and archaeology
☐	☒	Animals and other organisms
☒	☐	Clinical data
☒	☐	Dual use research of concern
☒	☐	Plants

Methods

n/a	Involved in the study	
☒	☐	ChIP-seq
☒	☐	Flow cytometry
☒	☐	MRI-based neuroimaging

Antibodies

Antibodies used	Rabbit anti-ACRV1 (Proteintech, 14040-1-AP)<br>Rabbit anti-AIF (Cell Signaling Technology, 5318)<br>Mouse anti-Actin-HRP (Sigma-aldrich, A3854)<br>Rabbit-anti-EEA1 (Cell Signaling Technology, 3288)<br>Rabbit anti-GM130 (Sigma-aldrich, G7295)

	Rabbit anti-Golgin97 (Cell Signaling Technology, 13192)<br>Rabbit anti-Hexokinase I (Millipore, Ab3543)<br>Rabbit-anti-PDI (Cell Signaling Technology, 3501)<br>Rabbit anti-PGK2 (Abcam, Ab183031)<br>Goat anti-PRSS21 (Thermo Fisher, PA5-47879)<br>Rabbit-anti-RCAS1 (Cell Signaling Technology, 12290)<br>Rabbit anti-SYPL1 (Abcam, Ab184176)<br>Rabbit anti-VAMP2 (Abcam, Ab181869)<br>Rabbit anti-VAMP3 (Abcam, Ab43080)<br>Rabbit anti-VAMP4 (Proteintech, 10738-1-AP)<br>Donkey Anti-Goat IgG H&L (Alexa Fluor® 555) (ThermoFisher, A21432)<br>HRP conjugated goat anti-rabbit IgG (Bio-Rad, 1706515)<br>HRP conjugated rabbit anti-goat IgG (Bio-Rad, 1721034)<br>Zenon™ Alexa Fluor™ 488 Rabbit IgG Labeling Kit (ThermoFisher, Z25302)<br>Zenon™ Alexa Fluor™ 555 Rabbit IgG Labeling Kit (ThermoFisher, Z25305)
Validation	All antibodies have been validated by vendors or previous publications. Detailed dilutions for each antibody were provided in the methods. Antibody specificity was evaluated using respective negative controls.

Animals and other research organisms

Policy information about studies involving animals; ARRIVE guidelines recommended for reporting animal research, and Sex and Gender in Research

Laboratory animals	The 8-20 week-old Sypl1 control and mutant mice in C57BL/6N background were used in this study. Since this study is focused on male reproduction, male mice were used for sample collection and female mice were only used as breeders to generate offspring of desired genotypes.
Wild animals	This study did not involve wild animals.
Reporting on sex	This study reports on sperm biology. We only used male mice for sample collection.
Field-collected samples	This study did not involve samples collected from field.
Ethics oversight	All mice were bred and housed under specific pathogen-free conditions with controlled temperature (20-25°C), 50-70% humidity and exposed to a constant 12-hour light-dark cycle in the animal facilities of Michigan State University. All the animal procedures were reviewed and approved by the Institutional Animal Care and Use Committee of Michigan State University. All experiments with mice were performed ethically according to the Guide for the Care and Use of Laboratory Animals and institutional guidelines.

Note that full information on the approval of the study protocol must also be provided in the manuscript.
