# Supplementary material for: SYPL1 defines a vesicular pathway essential for sperm cytoplasmic droplet formation and male fertility
Source: Nat Commun. 2023 Aug 22;14:5113. doi: 10.1038/s41467-023-40862-1 (PMC10444883; doi:10.1038/s41467-023-40862-1)
Supplement: Supplementary file 4 — Source Data [file 41467_2023_40862_MOESM4_ESM.zip › Source Data 2.pdf]

# Uncropped gel and Western blot

## Fig. 1d

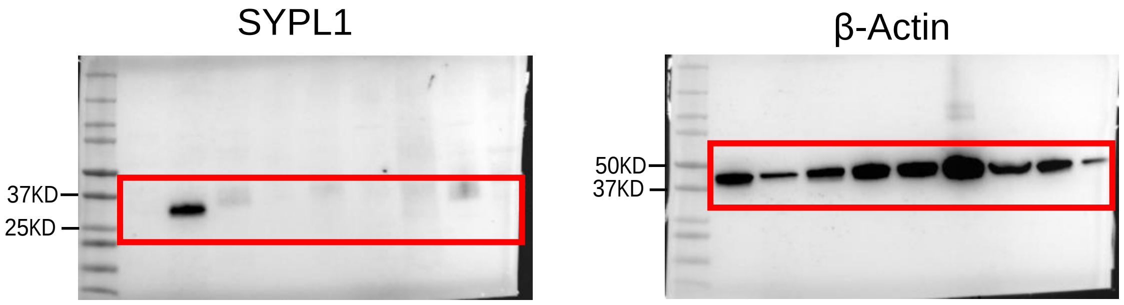

**Fig. 2b**

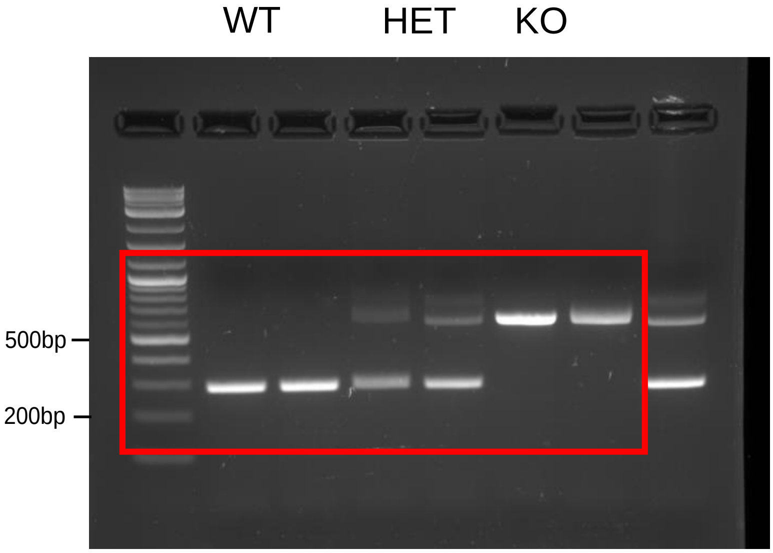
WT
HET
KO
500bp
200bp

**Fig. 2c**

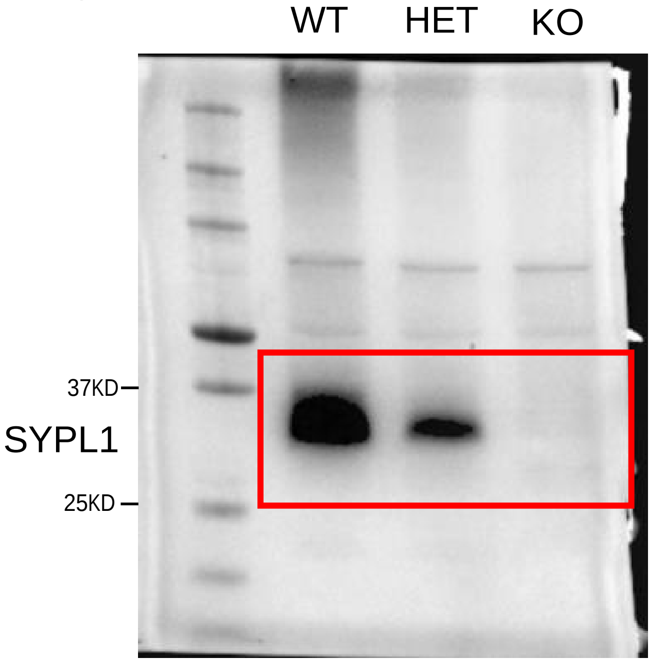
WT
HET
KO
37KD
SYPL1
25KD

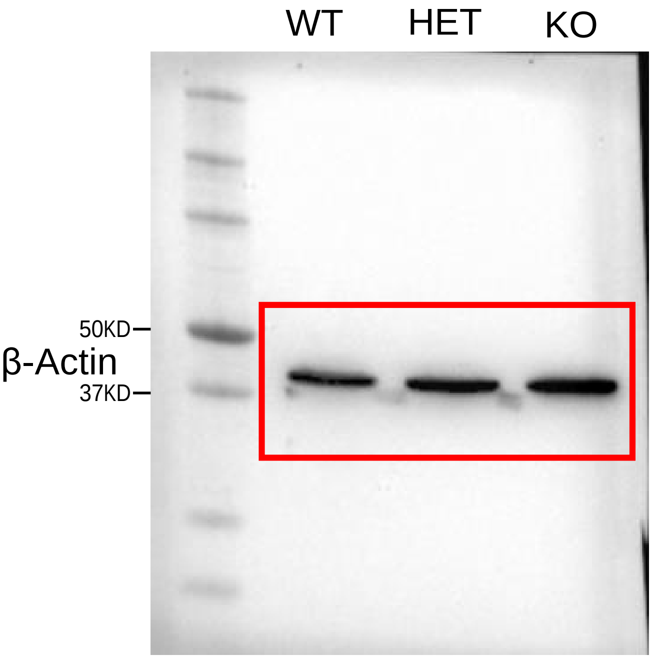
WT
HET
KO
50KD
β-Actin
37KD

**Fig. 3g** Testis

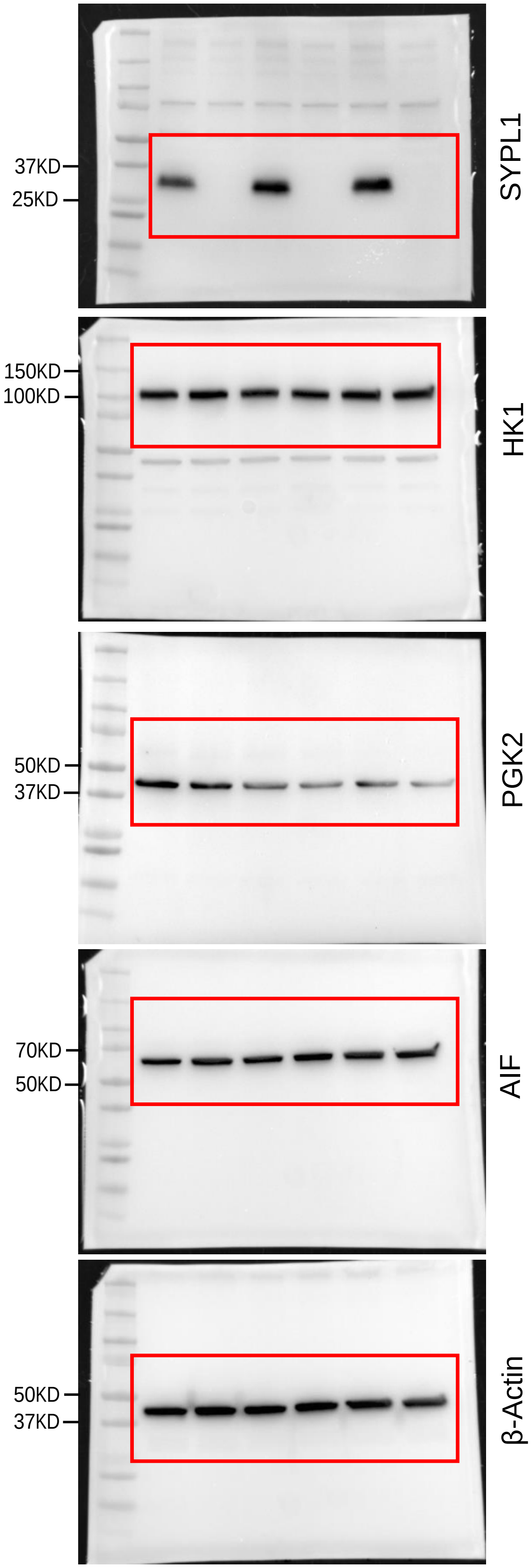


**Fig. 3h** Caput sperm

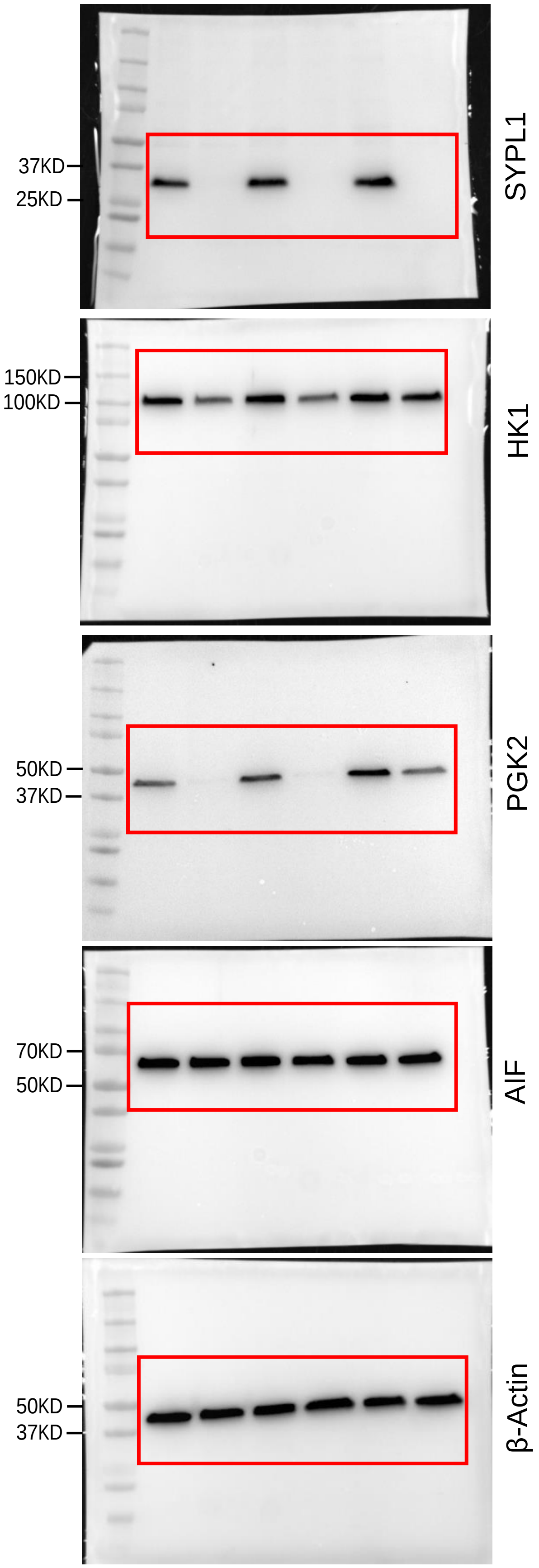

**Fig. 5b** **Fig. 5c**

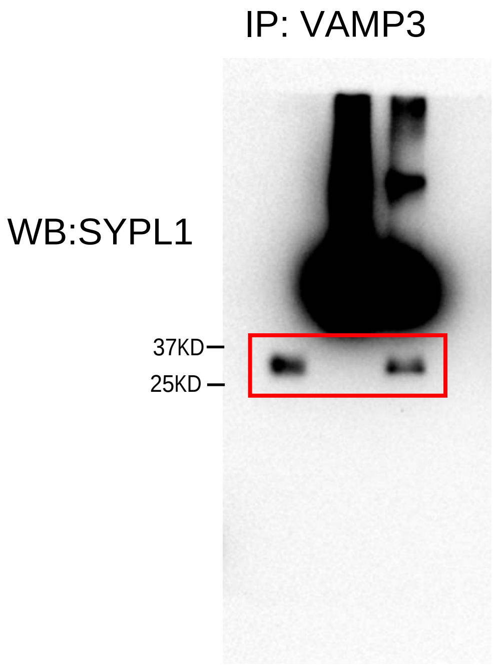


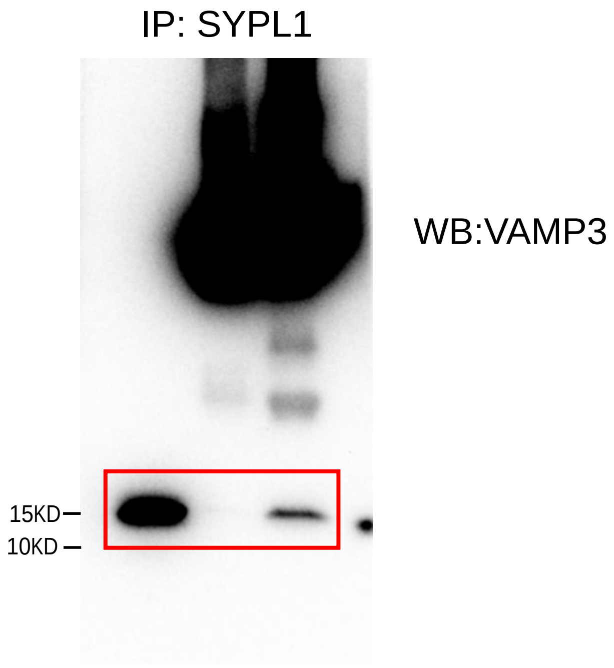

## Fig. S7a

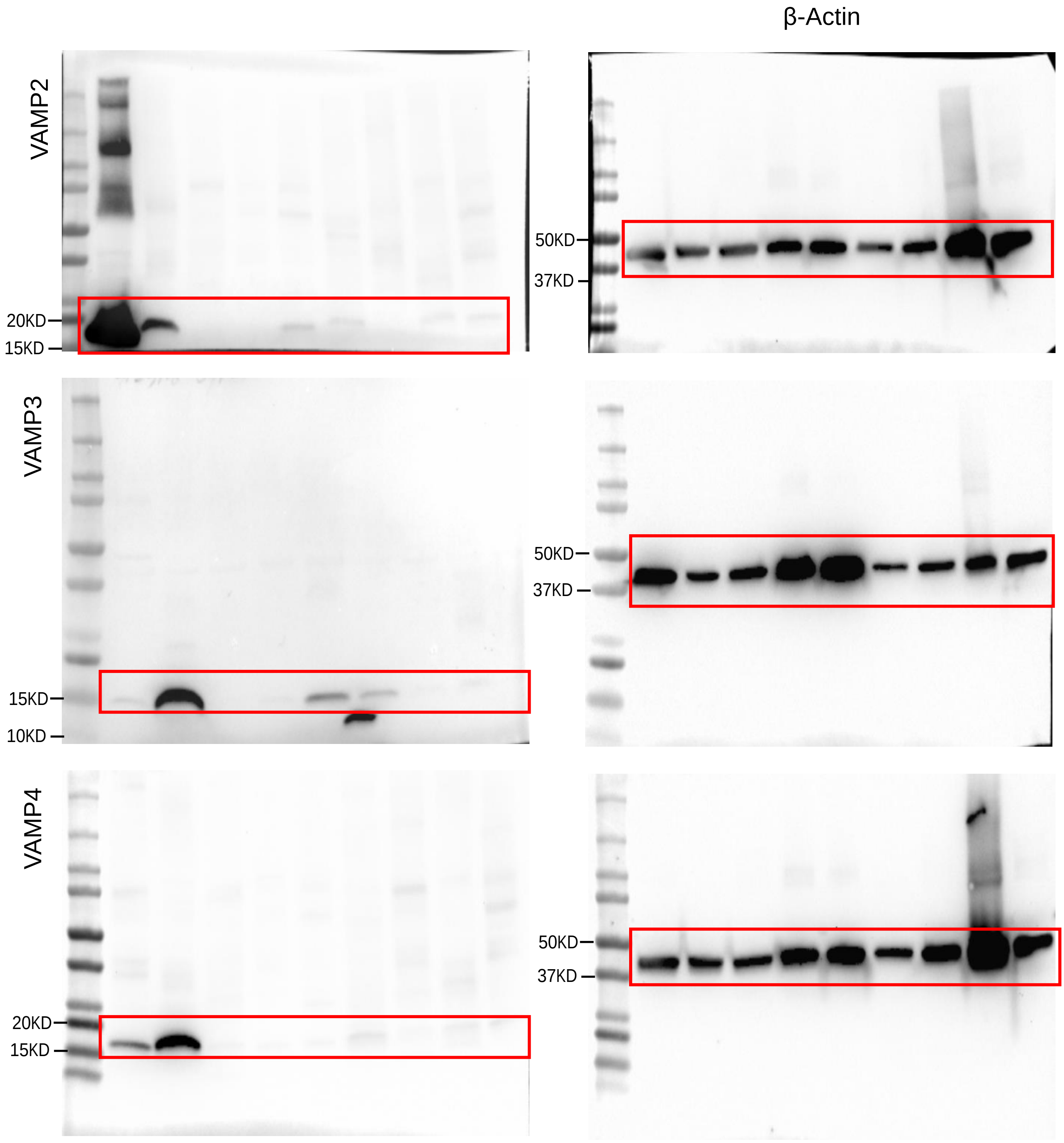

β-Actin
VAMP2
20KD
15KD
50KD
37KD
VAMP3
15KD
10KD
50KD
37KD
VAMP4
20KD
15KD
50KD
37KD

## Fig. S8a

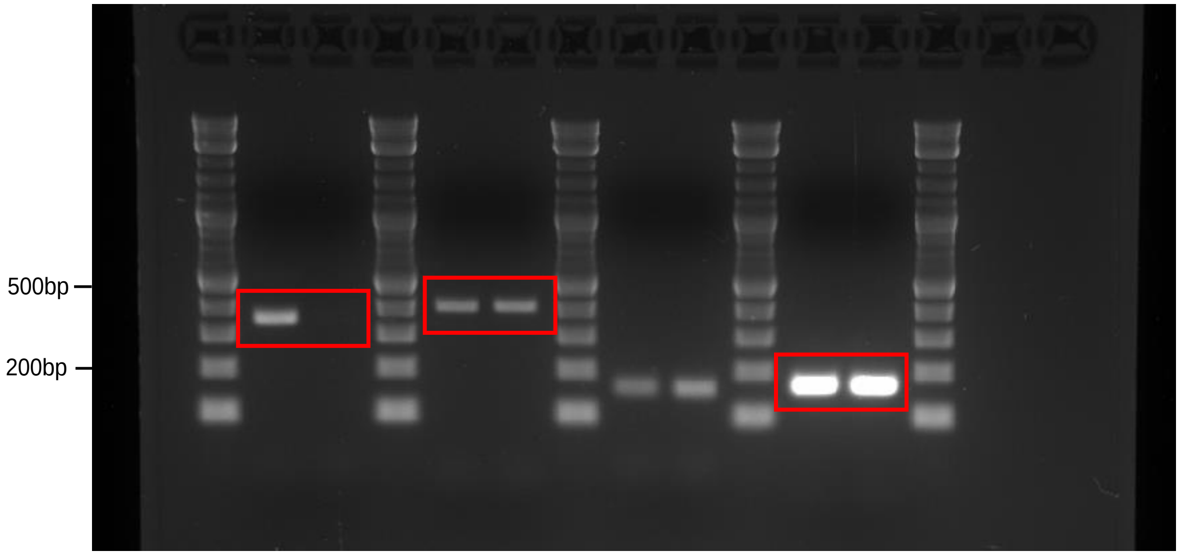


## Fig. S8c

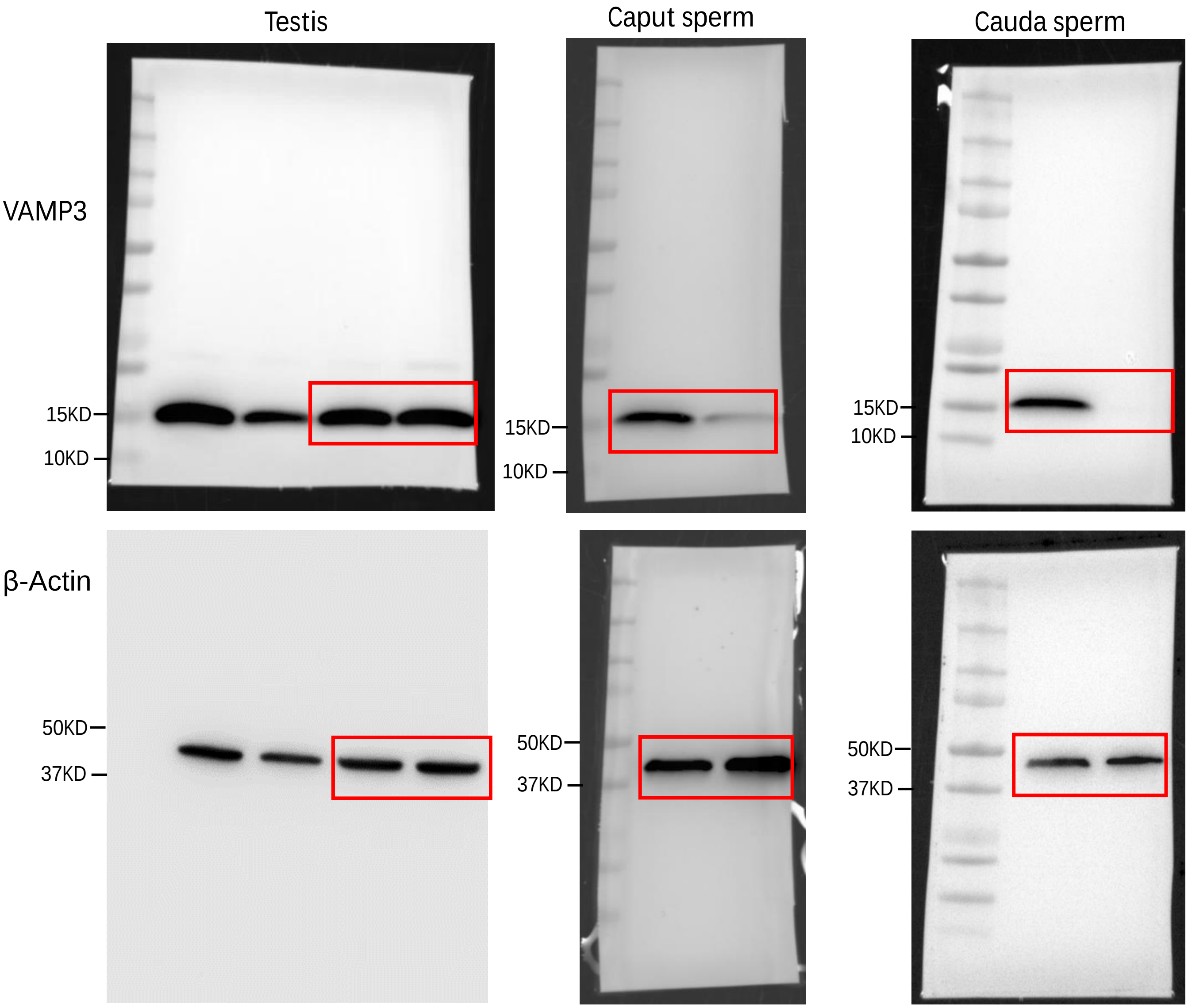

**Fig. S9b**

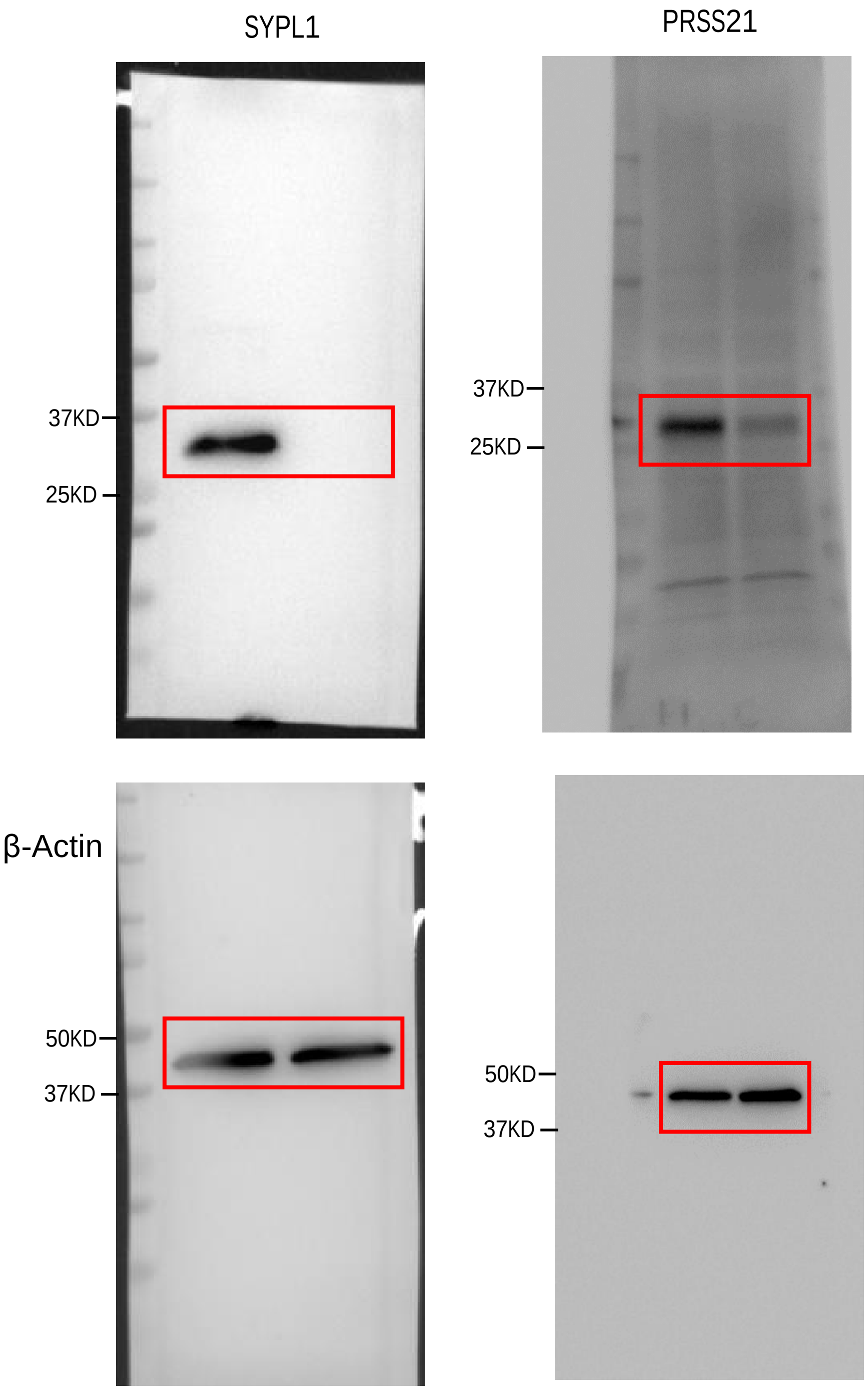

## Fig. S10b

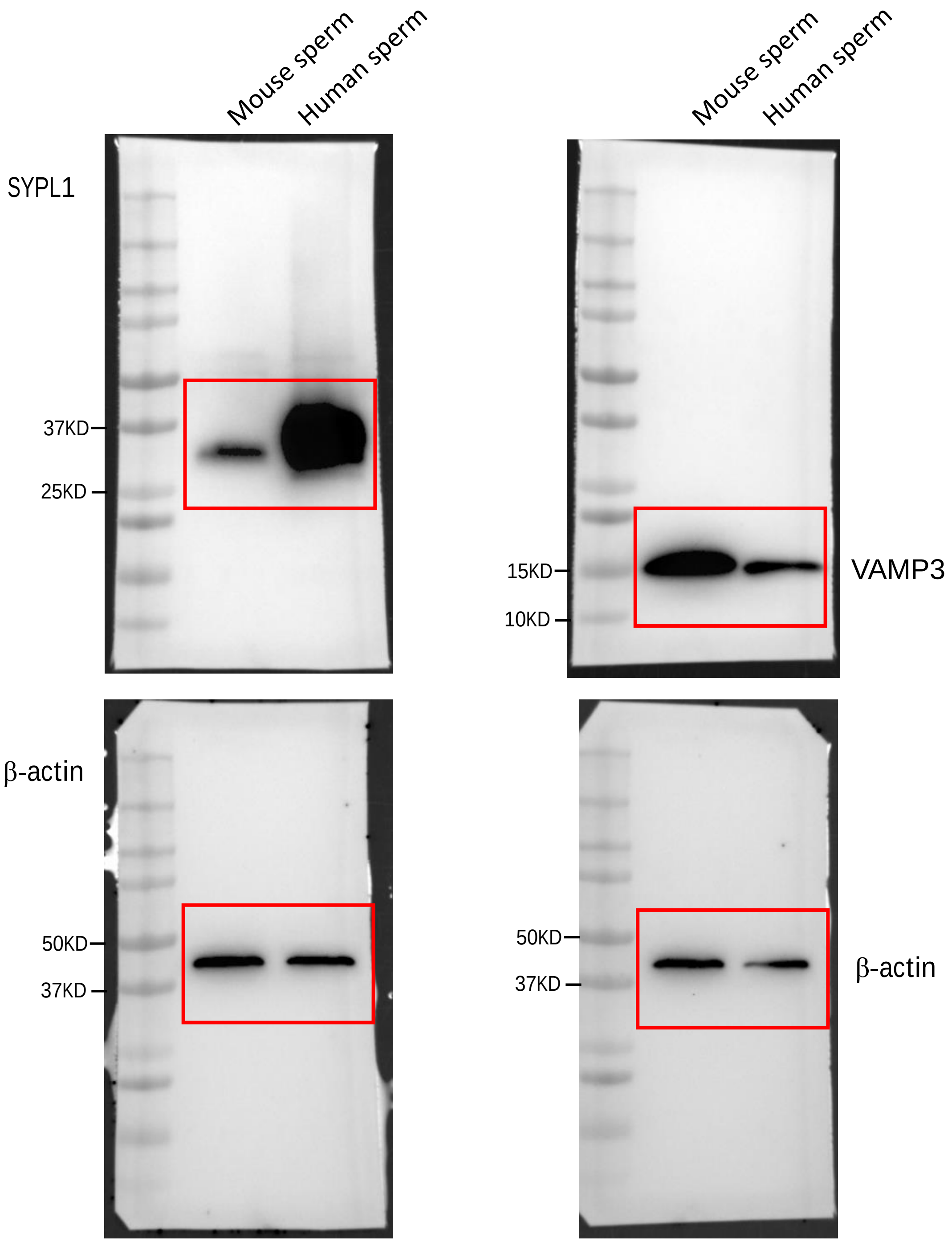

Mouse sperm
Human sperm
SYPL1
37KD
25KD
Mouse sperm
Human sperm
15KD
10KD
VAMP3
β-actin
50KD
37KD
50KD
37KD
β-actin
